# Simrank: Rapid and sensitive general-purpose k-mer search tool

**DOI:** 10.1186/1472-6785-11-11

**Published:** 2011-04-27

**Authors:** Todd Z DeSantis, Keith Keller, Ulas Karaoz, Alexander V Alekseyenko, Navjeet NS Singh, Eoin L Brodie, Zhiheng Pei, Gary L Andersen, Niels Larsen

**Affiliations:** 1 Ecology Department, Lawrence Berkeley National Laboratory, Berkeley, USA; 2 Physical Biosciences Division, Lawrence Berkeley National Laboratory, Berkeley, USA; 3 Department of Microbiology, New York University School of Medicine, New York, USA; 4 Department of Molecular Biology, Aarhus University, Aarhus, Denmark; 5 Center for Health Informatics and Bioinformatics, New York University Langone Medical Center, New York, USA

## Abstract

**Background:**

Terabyte-scale collections of string-encoded data are expected from consortia efforts such as the Human Microbiome Project http://nihroadmap.nih.gov/hmp. Intra- and inter-project data similarity searches are enabled by rapid k-mer matching strategies. Software applications for sequence database partitioning, guide tree estimation, molecular classification and alignment acceleration have benefited from embedded k-mer searches as sub-routines. However, a rapid, general-purpose, open-source, flexible, stand-alone k-mer tool has not been available.

**Results:**

Here we present a stand-alone utility, Simrank, which allows users to rapidly identify database strings the most similar to query strings. Performance testing of Simrank and related tools against DNA, RNA, protein and human-languages found Simrank 10X to 928X faster depending on the dataset.

**Conclusions:**

Simrank provides molecular ecologists with a high-throughput, open source choice for comparing large sequence sets to find similarity.

## Background

Molecular ecology methods often require the collection of thousands of polymer sequences (DNA, RNA or proteins) extracted from biological specimens (isolates or communities) followed by a similarity search of those sequences against one or more reference databases. The match results enable the deduction of community composition [1] or inference of functional capacity [2,3] within organisms or across populations. The most popular method for sequence comparison has been to find local alignment pairings using BLAST [4] but due to speed limitations, other software has emerged to bypass the time-consuming alignment step by simply counting the number of short sub-sequences shared between a subject and query. Sub-sequence oligomers are referred to as k-mers and are the set of possible fragments of a given length (2-mer, 3-mer, 4-mer, etc.) from a polymer. K-mer matching has been employed for diverse objectives in genomics including bacterial gene discovery [5], identifying DNA signatures of pathogenic bacterial genomes [6], delineating plant genome polyadenylation sites [7], spotting genetic engineering in bacteria [8], assembling shotgun DNA sequences [9], human genome re-sequencing [10], protein superfamily recognition [11], and sequence clustering [12]. Rapid k-mer similarity searches have become the foundation for high-throughput phylogenetic classification of DNA [13-15]. Surprisingly, a general-purpose open-source software tool to aid biologists in performing all the aforementioned tasks is not readily available. MICA [16] can match DNA k-mers against a genome but requires a Windows or Macintosh GUI, is not open source and is restricted to 7-mers or shorter. SSAHA2 [17] is less limited but is impeded by coupling k-mer searching with non-optional local alignments that are unnecessary for some applications. Unfortunately, SSAHA2 does not search protein sequences. Cd-hit [12] efficiently evaluates k-mer set unions for the purpose of single-linkage (nearest-neighbor) clustering. Cd-hit does not allow the decoupling of k-mer searches from the clustering, thus it is not used as a general-purpose similarity reporting tool.

Simrank was conceived to avert these limitations. The earliest version (N. Larsen, unpublished) was produced to run as a web service for the Ribosomal Database Project, starting in 1992 [18]. It was coded in FORTRAN when only a few hundred 16S rRNA gene sequences had been determined, and was able to index a maximum of 33,000 sequences. Since FORTRAN popularity has generally waned in comparison to PERL and C [19], Simrank was reimplemented to encourage greater community involvement and extended for usage with larger datasets. The PERL/C implementation described here has a database limit of 2 billion sequences, but this limit can be lifted by changing constants within the source code. Compared to the alternatives, Simrank is the only choice that is completely open source, quickly estimates the overall similarity between query and database sequences, compiles and runs on all contemporary hardware and operating systems, is sans GUI allowing pipeline integration, eschews sequence alignment and clustering steps, allows user-definable search depths, is unrestrictive of k-mer sizes, and is unrestrictive of polymer or string type. If sequences can be represented as text strings, such as nucleic acids, proteins, and even human-readable language, then they can be quickly compared using Simrank.

Simrank has enabled advances in curation and annotation practices of large biomarker data-sets such as the Greengenes 16S rRNA gene database [13] and has aided in creating guide-trees, OTUs and probe performance predictions for the PhyloChip™ assay (Second Genome, San Francisco, CA)[20]. Microbial ecologists have employed Simrank to annotate 16S rRNA gene sequence libraries by comparisons to reference databases [21-23]. Counts of sequences matching each taxon are used as proxies for community structure and are compared across clinical or environmental samples by researchers to elucidate niche effects such as competition, selection, resource partitioning and colonization [24]. Simrank's utility to molecular microbial ecologists will continue to grow concomitant with the size of sequence datasets.

## Implementation

Simrank is implemented mainly as an object-oriented PERL module, with one 5-line function written in C for efficiency. An example script is included with the software which allows parameter choices for many features directly from a command line. Accessing the object directly within a PERL program allows all features to be parameterized.

### Inputs

The input files (reference database or query set) are FastA formatted multiple sequence files and do not need to be aligned. For each record only two newline-separated fields are required, the header and the string itself. The header begins with the ">" character and can contain any number of fields separated by characters convenient for the user's work flow. The one constraint, is that within the header must be a unique string identifier between the ">" and the first space or newline. For example, within the header ">gg_id244724 cattle rumen clone YNRC11\n", "gg_id244724" is considered the unique identifier. Following the header is the string itself which can be DNA, RNA, protein, human readable language or other text.

### Database Format

From the input, a binary file is generated optimized for retrieval of k-mer similarities. The binary file contains a pre-computed map between all unique k-mers and a list of all sequences containing that k-mer. Recorded k-mers can be restricted to those entirely composed of a user-defined alphabet (e.g. ACGT for DNA databases).

### Formatting procedure

Each string is assigned an integer index and then is split into all valid k-mers of user-defined lengths (default 7-mers) and alphabets. The k-mers are overlapping substrings representing the contiguous source string. Unique k-mers are hashed and counted. Each k-mer is associated with an array of offsets representing all the string indices containing the given k-mer. Specifically, each integer in this ordered list is the number of indices to skip to find the next string index containing this k-mer. This information is encoded in a binary file ordered according to Table 1.

**Table 1:** Simrank database binary file structure and storage requirements.

File Segment	File Element	Storage Requirement (bytes)
1	F, string ID field size	10
2	K, k-mer length	10
3	N, string count	10
4	string ID array	*FN *
5	offset arrays^a ^	
6	k-mer array^b ^	*Kk *
7	offsets index array^c^	4*k *
8	offsets lengths array^d^	4*k *
9	unique k-mers per string array^e^	4*N *
10	k, unique k-mer count	10
11	file position of segment 6	10

^a^Each k-mer generates a vector of string indices, encoded as an integer array of offsets required to "visit" each string index containing the k-mer. k is the count of unique k-mers, and s_i _is the count of strings containing the i^th ^k-mer. Each offset is stored as a 4-byte integer.

^b^Lexically sorted ASCII text strings of each unique k-mer stored as one byte per character.

^c^4-byte integer list of file positions for the start of each k-mer's list of offsets.

^d^4-byte integer list of the byte length of each k-mer's list of offsets.

^e^4-byte integer list of the count of unique k-mers in each string.

### Search procedure

Simrank's search procedure is initialized by reading minimal database attributes into memory. Then, query strings are handled serially to calculate similarity to each database string. In the initialization, six of the eleven database file segments (Table 1) are read: the list of string identifiers, k-mer length, all unique k-mers, counts of unique k-mers in each string, and the file's start positions and lengths of each k-mer's offset array. Constraining disk access to only these elements minimizes pre-search lag-time. An in-memory PERL data structure is established as a hash of k-mer keys, each referencing two pointers, the begin byte position of list of offsets and the length of the offset. Since the database file structure is governed by the k-mer length, each unique combination of a reference string file and k-mer length will require its own database creation.

Each query string initializes a C scoring vector of length equal to the number of strings in the database × 4 bytes. All scores are set to zeros. Next, Simrank extracts all unique query k-mers according to user-defined length and alphabet restrictions and sorts them lexically. Any query k-mer found in the database begins a file seek to read the list of sequence id offsets allowing increments of scores for corresponding elements in the scoring vector. Lookups and increments occur in precompiled C routines. After all query k-mers are examined, Simrank returns a sorted list of similarities as a table. The similarity between sequences Q and S are the number of unique k-mers shared, divided by the smallest total unique k-mer count in either Q or S.

## Results

**Figure 1:**
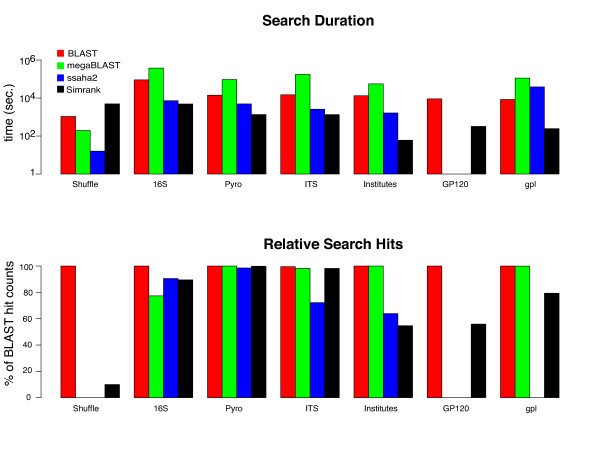
**Search duration and relative hit results**. Comparison of search duration and hits between search tools with various data sets. Data sets are described in Table 2. Search duration is expressed in seconds and shown in log scale. Hit results are expressed as a percentage in relation to subject hit counts from BLAST's local alignments.

Diverse data sets (DNA, RNA, protein and human readable language) of various sizes ranging from 2.8 million to 687 million characters (Table 1) were used for testing Simrank in comparison to other tools (Figure 1). Simrank was able to index each dataset according to various k-mer and alphabet sizes. SSAHA2 and megaBLAST were unable to index the protein dataset. Language indexing and searching was tested using institute names extracted from GenBank records. Indexing the list of institute names directly was impossible for SSAHA2, BLAST and megaBLAST, so an artificial conversion from language to DNA [25] was performed.

Simrank considered *all *regions of both the query and database sequences in each pair-wise calculation of similarity. Since BLAST constrains its results to *only *sub-regions of high similarity, it was run with parameter '-q -1' to allow longer match regions and equitable comparison to Simrank. BLAST was accelerated with parameters '-S 1 -e 0 -m 8' so that only the top strand was searched for significant matches and minimal disk writes were required. In all comparison tests, the top ten database matches with over 90% identity (as defined by each program) were requested for each query string except for BLAST where percent identity thresholds are not definable. All software comparisons were conducted on a Linux server with Dual-Core AMD Opteron 8216 2.4 GHz processors and 32 GB of shared memory but Simrank does not require hardware with large memory. For example, a MacOSX laptop with an Intel Dual-Core i5 2.4 GHz processor required only 66 MB of memory and completed the ITS test (Table 2) in 540 seconds (similar to the speed observed on the 32 GB server). Figure 1-top displays the log-scale time required to complete each search. Simrank completed its search in less time than all other tools in all dataset types. The only exception was in the randomly shuffled DNA dataset test where SSAHA2 completed its search before all others. Search hit counts were measured in comparison to BLAST. The number of query-subject relationships each tool returned was divided by the number returned from BLAST. Since BLAST constrained hits by e-values equal to 0, hits with under 90% similarity were counted resulting in BLAST returning the majority of hits across all datasets. Figure 1-bottom reveals comparable hit counts among the tools for real DNA datasets such as the 16S rRNA database, the pryrosequence library and the ITS database (Table 2). The shuffled (synthetic) DNA library was included in the dataset as a negative control where only insignificant hits are expected. Simrank reported fewer of these insignificant hits than BLAST but megaBLAST and SSAHA2 ignored them all.

**Table 2:** Datasets used for performance evaluation

Data Set	String Type	Mean Length	Database Count	QueryCount	alphabet size	k-mer length	total database k-mers
16S^a^	DNA	1350	188,073	2000	4	7	16,384
Pyro^b^	DNA	150	501,532	500	4	6	4,096
ITS^c^	DNA	627	212,367	2000	4	6	4,096
Shuffle^d ^	DNA	687	1,000,000	1000	4	7	16,384
gpI^e^	RNA	398	20,085	5000	4	7	16,360
GP120^f^	Protein	175	68,119	2000	20	4	98,695
Institutes^g^	Text	121	23,768	1000	47/61	4	67,287

^a ^Greengenes 16S rRNA gene collection (DeSantis, 2006)

^b ^Roche-454 pyrosequences from gastrointestinal contents (Ochman, 2010)

^c ^Internal Transcribed Spacer region from eukaryotic ribosomal genes.

^d ^Derived from random repetitive shuffling of *Ralstonia solanacearum *strain UW486 endoglucanase precursor, DQ657652 (Castillo and Greenberg, 2007)

^e ^Group I catalytic introns RFAM RF00028 (Griffiths-Jones, et al., 2003)

^f ^HIV Envelope glycoprotein PFAM PF00516 (Finn, 2008)

^g ^Institute names as displayed in GenBank records. For BLAST and SSAHA2, all non-alphanumeric characters were interpreted as a space for a total of alphabet size of 47, for Simrank no substitution for any of the 61 unique characters was performed.

The protein and RNA datasets revealed a large contrast among the tools. Only Simrank and BLAST were able to search protein sequences and BLAST returned the greatest number of hits given the constraints. RNA searches were possible with all tools but SSAHA2 was unable to find matches and Simrank found less than both BLAST and megaBLAST.

The institute affiliation data set was comprised of character strings representing over 23,000 academic departments and addresses found in GenBank records. Simrank was able to not only find exact matches but also to rapidly detect highly similar inexact matches. For instance, "Institut National de la Recherche Agronomique, Avenue des Etangs, Narbonne 11100, France" and "Laboratoire de Biotechnologie de l'Environnement, Institut National de la Recherche Agronomique, Avenue des Etangs, Narbonne 11 100, France" shared 96.47% of their 4-mers. The BLAST tools and SSAHA2 were effective at finding these relationships as well but only after the artificial conversion [25] from language to DNA.

**Figure 2:**
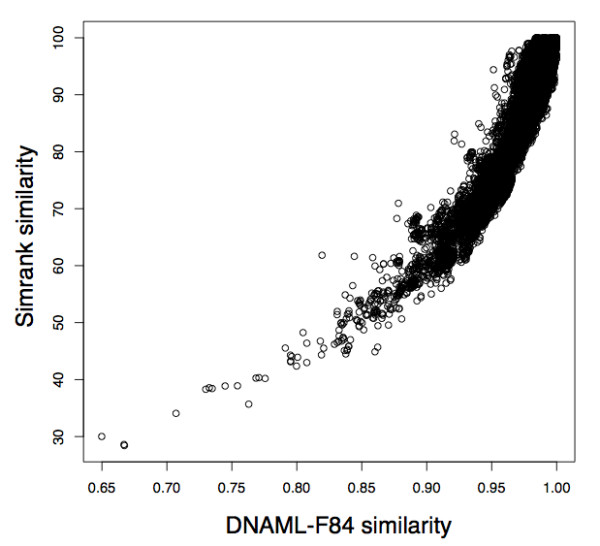
**Similarity score comparison**. Comparison of DNA sequence similarity scores observed when a single DNA sequence collection is compared to a reference database using either Simrank or an alignment-based scoring system.

Comparison of an experimentally obtained 16S rRNA gene library [26] against a 16S reference set is plotted in Figure 2. Similarities were calculated using Simrank with 7-mers or the alignment-based F84 similarity, a metric commonly used as a measure of phylogenetic divergence [27-29]. Each circle represents a single sequence. The majority of observed pairings exceeded 90% by F84 distance and 60% by Simrank distance.

**Figure 3:**
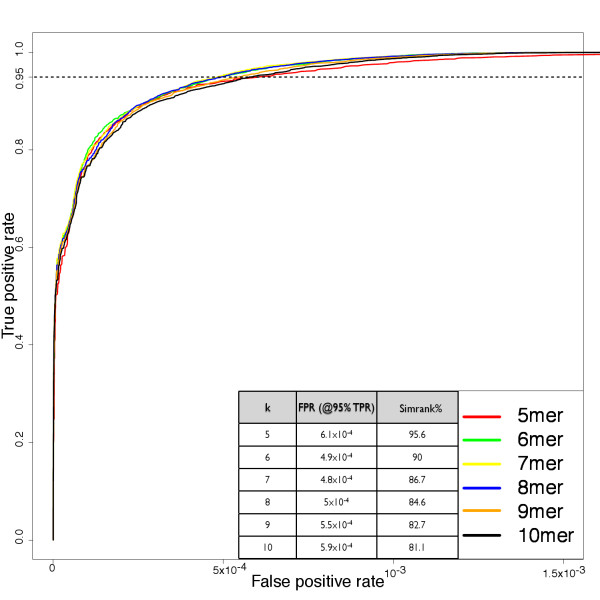
**Simrank sensitivity and specificity**. Comparison of sensitivity and specificity of Simrank DNA searches with various k-mer lengths. True hits were defined as those with 97% alignment identity. The x-axis is the false positive rate (FPR - Simrank hits to subjects with <97% alignment identity), the y-axis is the true positive rate(TPR - Simrank hits to subjects with > = 97% alignment identity). Each curve represents the balance of TPR and FPR through the range of Simrank thresholds. Vertical dashed line at y = 0.95, represents a 95% TPR. Inset table lists the FPR and Simrank cutoff for each k-mer search to obtain a 95% TPR.

In a test of DNA search sensitivity, 50 16S rRNA gene queries were drawn randomly from the Greengenes set of 188,073 subjects. All query-to-subject full-length alignments were found with BLAST (-q -1) and were recored whenever the percent identity was >= 97%, calculated as *i*/min(*L*_q_, *L*_s_), where *i *is the count of pair-wise base identities and *L*_q _and *L*_s _are the lengths of the query and subject strings, respectively. These recorded matches were labeled as "true positives" for reference in Receiver Operator Characteristic (ROC) analysis [30]. The same 50 queries were Simrank compared to all subjects using multiple k-mer lengths (from 5-mers to 10-mers) and the results are presented in ROC curve format as Figure 3.

### Memory

The memory consumption of Simrank during indexing is moderate and grows linearly with the number of sequences and depends on the k-mer size defined by the user. For example, when the 16S data set containing sequences with a mean length of 1,350 characters was indexed on 7-mers, 50 MB of memory was utilized for every 20,000 sequences.

## Discussion

As expected, Simrank was able to search bio-polymer databases in less time than local alignment search tools. Simrank was 10X to 928X faster than the BLAST tools in finding similarities among DNA, RNA and proteins. The rapid delivery of results is enabled by the simplistic calculation requiring no bottleneck alignment steps. Since SSAHA2 employs a hybrid strategy of building pair-wise alignments but only against records achieving significant k-mer identities, it was expected to exhibit speeds between BLAST and Simrank. This prediction was observed in Figure 1-top where Simrank is shown to be only 1.5X to 158X faster than SSAHA2 when tested against public DNA and RNA datasets. SSAHA2 was unable to search protein databases. Simrank and BLAST lagged behind megaBLAST and SSAHA2 when searching shuffled DNA sequences (i.e. synthetic dataset), but were able to find distant relationships missed by the others. SSAHA2 and megaBLAST require larger seeds to elicit alignments and thus searches terminated quickly. Conversely, Simrank and BLAST examined each 7-mer in each query requiring more compute time but enabling distant similarity reporting.

The method of hit count measurement displayed in Figure 1-bottom presents serious drawbacks. Similarity scales across the tools are not strictly equivalent (as noted in Figure 2 and in "Usage Considerations"), therefore, a 90% match has not the same meaning in Simrank as it may have in the context of an alignment-based score. Comparison of different scales with a uniform threshold does not convey the true sensitivity of Simrank. In order to more directly address the question of sensitivity, a test was conducted to determine the ability of Simrank to find homologues with 97% identity, a popular cutoff for Operational Taxonomic Unit (OTU) boundaries used in molecular microbial ecology [20]. Figure 3 demonstrates the capacity of Simrank's similarity measure to find appropriate database subjects with a reasonable number of false positives and false negatives despite the difference in scoring scales. This approach allows calibration of Simrank and definition of appropriate thresholds. For example, to find query-subject pairs with 97% full-alignment identity within the 16S dataset, one could utilize a Simrank k-mer size of 8 and score threshold of 84.6% to realize a true positive rate of 95.00% with a corresponding false positive rate of just 00.05%. This means that Simrank matches with over 84.6% 8-mer identity will cover 95% of the BLAST hits but will also match a very small number of strings not found by BLAST.

Although not included in the Figure 1, we observed that BLAST and SSAHA2 database formatting procedures are faster than Simrank's. For this reason we suggest using BLAST or SSAHA2 for exploratory sequence comparison since trial-and-error databases can be created and destroyed rapidly, but to select Simrank for persistent datasets where various queries will be compared to a fixed set of sequences. Consequently, the Greengenes web service [13] utilizes Simrank as the search engine for sequence comparison and taxonomic classification of arbitrary user sequences against a reference data set.

Simrank can run in stand-alone mode or as a PERL module within a simple or complex pipeline. The components are modular so various phases of a pipeline can separately encode databases, initialize search factories in memory, and/or process queries as batches or data streams. Simrank accepts user parameters to filter results by depth and/or percent similarity. This is an advantage in high-throughput environments over BLAST, for instance, since post-processing filtering scripts are not needed.

Simrank may allow recovery of useful information from error-laden sequences. A current problem in the popular pyrosequencing technique is the reporting of long homopolymers not verifiable by traditional sequencing techniques [31]. Simrank eliminates the effect of sequence discrepancies arising solely from homopolymer exaggeration. For instance, a run of 7 consecutive A's can be recorded as one unique 6mer. Thus, if the only polymorphism differentiating two query sequences is the length of an unsubstantiated homopolymer, their Simrank scores against a database will be equivalent.

While this manuscript was under review, another k-mer leveraging software package, UCLUST/SEARCH [32] was published. Although it is not open-source and requires a paid license for 64-bit versions or commercial use, it does have potential to be highly useful for rapid k-mer searches as well as sequence alignments.

### Usage considerations

From observations summarized in Figure 1, it is advised that Simrank is not suitable for searching randomly shuffled DNA, marginally suitable for matching proteins or strings of highly variable content such as group I self-splicing introns where similarity is limited to only two short spans [33]. Simrank is well-suited for searching variants of full-length homologous strings such as 16S rRNA genes, partial-length homologous strings such as those created by Roche-454 sequencing technology, and variants of eukaryotic internal transcribed spacer regions.

Simrank similarity scores are not equivalent to alignment percent similarities. For example, Figure 2 displays differences in similarity scores observed when a single DNA sequence collection [26] is compared to a reference database using Simrank versus the alignment-based F84 scoring distance [27]. Alignment identities of 90% can produce Simrank identities of 55-70%, and conversely, Simrank identities of 90% can produce alignment identities of 93-99%. The differences are caused by two factors. First, one sequence may contain repetitive k-mers at disjointed positions leading to a perceived increase in similarity, and second the spatial distribution of mismatches can lead to divergence of Simrank and BLAST scoring. For example if every 1 in 7 bases are mismatched in a pair-wise alignment, then Simrank using 7-mers would report a 0% similarity where BLAST would conclude 86% similarity. Thus, tuning k-mer length to the expected frequency of mismatches may result in application-adapted search sensitivity.

Levels of significance for hits to protein sequences should be established based on known reference sets. Protein strings are generally shorter than gene strings and their similarity patterns are often single conserved amino acid positions separated by one or two variable positions. The search for 4-mer similarities within the GP120 protein dataset revealed this difficulty. The BLASTp alignment procedure, although 28X slower, was nearly twice as sensitive compared to Simrank.

Furthermore, since each k-mer is compared across sequences without regard to their relative position in the sequences, Simrank is insensitive to continuous and non-continuous patterns within the sequence such as sites of potential secondary structure. As with all inter-sequence comparisons, search results decline in significance when comparing a very short versus a long sequence. Users can set lower length limits to avoid misleading match pairs.

As noted in Table 2, the language search comparison encountered 61 unique characters in the institute names but the complexity was reduced to 46 characters for BLAST and SSAHA2. BLAST and megaBLAST were able to find twice as many matches than Simrank but the significance of these hits are questionable since BLAST's local alignments allow one word such as "University" to produce high-scoring pairs. Of the tools, only Simrank tested the entire string for similarity.

Simrank search results across databases composed of strings with repetitive elements can be refined by setting the k-mer length to exceed the repeat length. Any repetitive k-mers within a string are counted only once since only the unique counts are used to create the quotient. In this case, Simrank percent similarity scores would be inflated relative to BLAST.

### Future work

Common tasks in molecular microbial ecology may be facilitated with Simrank. Applications include dataset de-replication, sequence clustering, and rapid classification. In upcoming versions, we plan to provide options to reduce database file sizes and memory requirements for constrained alphabets. For instance non-ambiguous DNA can be encoded with 2 bits for each base instead of 8. To further increase speed during batch queries, a non-redundant strategy will be made available allowing a pre-screen of the batch to identify all unique k-mers before reading offset arrays from disk. This will prevent common k-mers from inducing repetitive file reads. Because strings within biological query sets can often contain similar k-mers, we estimate a >5-fold speed increase. To increase the ability to filter hits from a large databases of various length strings, a significance score can be added which considers the likelihood of a percent similarity score given the number of total unique k-mers in the query-subject comparison. This feature will generally down-weight matches from short strings compared to long strings with equivalent percent k-mer identities. Lastly, Simrank can be extended to store and output the string coordinates where k-mers match, should that become desirable. The computationally intensive k-mer tally procedure was written in C for speed but the IO and formatting is written in PERL for easy adaptations and extensions by computational biologists. It is the authors' intentions that other bioinformaticians will be able to improve the open source code where necessary to meet the needs of their projects. Please contact us if you would like to have your changes reflected in the distributed version.

## Conclusions

Simrank provides molecular ecologists with a high-throughput choice for comparing large sequence sets to find similarity. The software presented is orders of magnitude faster than its open-source counterparts, sensitive to low-similarity matches when desired, and flexible to allow similarity comparison for DNA, RNA, proteins and even written language. Simrank is specifically designed for matching queries against large reference sets. Two of Simrank's beneficial attributes are its speed and flexibility. It is capable of reporting significant hits faster than both BLAST and SSAHA2, moreover, Simrank is more flexible than CDHIT since k-mer searches are de-coupled from clustering.

## Availability and requirements

**Project name**: String::Simrank

**Project home page**: http://search.cpan.org/perldoc?String::Simrank

**Operating system(s)**: Platform independent

**Programming language**: PERL, C

Other requirements:

**License**: PERL Artistic License

**Any restrictions to use by non-academics**: No

## Abbreviations

MICA: k-Mer Indexing with Compact Arrays; SSAHA2: Sequence Search and Alignment by Hashing Algorithm; GUI: Graphical User Interface - the point-and-click requirements to operate a program; OTU: Operational Taxonomic Unit - a set of highly similar genes believed to carry phylogenetic relatedness; PERL: Practical Extraction and Report Language; ROC: Receiver Operator Characteristic - graphical plot of the sensitivity, or true positive rate, vs. false positive rate for a binary classifier system as its discrimination threshold is varied.

## Authors' contributions

NL conceived the indexing scheme and developed the original FORTRAN implementation and the original PERL/C implementation. TD forked the String::Simrank standalone PERL/C module, conducted the performance evaluation, created the Simrank web service and drafted the manuscript. GA, EB, and ZP specified the scaling requirements for the application and made critical revisions of the manuscript. KK and NS administrated the servers used to develop and test the module. NS and UK conducted testing with DNA data sets. UK and TD performed the ROC analysis. AA packaged the CPAN distribution. All authors reviewed and approved the manuscript.
